# Editorial: Healthcare Simulation and Online Learning

**DOI:** 10.3389/fsurg.2022.944020

**Published:** 2022-06-10

**Authors:** Zaleha Abdullah Mahdy, Ismail Mohd Saiboon, Dinker R. Pai, Michelle A. Kelly

**Affiliations:** ^1^ Department of Obstetrics and Gynaecology, Faculty of Medicine, Universiti Kebangsaan Malaysia, Cheras, Malaysia; ^2^ Department of Emergency Medicine, Faculty of Medicine, Universiti Kebangsaan Malaysia, Cheras, Malaysia; ^3^ Medical Simulation Centre, Mahatma Gandhi Medical College and Research Institute, Sr Balaji Vidyapeeth, Puducherry, India; ^4^ School of Nursing, Curtin University, Perth, Western Australia

**Keywords:** healthcare simulation, online learning, research topic, technology in education, interprofessional education

**Editorial on the Research Topic**

***Healthcare Simulation and Online Learning***

Healthcare simulation, delivered in multiple formats inclusive of online learning, has become more important than ever with the changing perceptions among both healthcare professionals and patients about how healthcare training should be conducted, and the central focus on patient safety and clinical competence. The idea of a research topic on healthcare simulation and online learning was first mooted following the inaugural conference of the Malaysian Society for Simulation in Healthcare (MaSSH) in March 2018, seriously pursued after the second MaSSH Conference in July 2019, and submitted to Frontiers in Surgery in 2020. However, in 2020 the COVID-19 pandemic struck, hence lending more weight to virtual simulation-based training, as well as swinging the pendulum of teaching and learning in all fields towards the online platform. We were therefore curious to know how this has impacted healthcare training in various localities globally. In fact, it was pertinent to reflect on strategies that were emerging due to the COVID-19 pandemic.

An editorial team consisting of individuals from different countries was assembled. The team moved through the task and communicated online managing the different time zones, an approach that reflected the theme of this special issue. The research topic was open to all scholars and clinicians, and submissions from numerous professions were received. A total of 32 manuscript submissions were submitted, out of which 15 were finally accepted, hence the work is now available as an eBook. As of 14 May 2022, the number of online views has exceeded 33,900 inclusive of North America, United Kingdom, Europe, and Asia, clearly demonstrating the popularity and global interest in the topic [Healthcare Simulation and Online Learning | Frontiers Research Topic (frontiersin.org)].

The response to this research topic has been an eye opener, as seen from the statistics presented earlier. Even though we have received a record number of submissions and online views, what is especially encouraging has been the diversity of the submissions. This diversity is in the form of article content, and the geographical regions from where the submissions have arisen, mirroring the diverse backgrounds of the guest editors of this research topic. It is heartening to note that many submissions have come from the Asian continent, which is a step in the right direction to make the topic more inclusive. This is also likely to have a spin off effect for the other journals in the Frontiers stable.

To illustrate this point, in terms of geographical distribution there were eight articles from Asia, four from Europe and one each from the United States, Brazil and Australia. In terms of article content, topics ranged from healthcare education to patient education and technology in simulation. To highlight a few, Valentim and co-authors looked at the value of online educational strategies for educating healthcare workers on the specific issues which impacted the health of prisoners in Brazil’s correctional system. Mohamad et al looked at how ontology could be used to explore the process of virtual reality (VR) simulator development and utilization. Mockrezecki and co-authors incorporated pharmacy faculty for training final year medical students, a classic example of interprofessional education (IPE), showcasing the benefits of learning together and sharing perspectives in order to understand and consolidate teamwork. The principle of building awareness of others’ roles, specialty knowledge and contribution to patient care was explored through a pilot study, which demonstrated that through simulation role-play, medical students’ prescribing skills improved significantly with pharmacist-led education. This represents a solid foundation for expanding such an approach to larger groups and highlights its potential to be formally included into future curricula. Another perspective was the utilization of simulation-based education (SBE) in conducting a low-fidelity low-cost in-situ neonatal resuscitation program (Pong et al). The approach was well received by major stakeholders, leading to funding of this type of training, which is well suited to low resource settings. From not knowing whether we would get enough articles for this research topic, to having the pleasure of such a broad tapestry of articles to review, has been a profoundly enriching experience. This reflects the growing uptake and broad applicability of contemporary technologies in health professions education and clinical training.

The sophisticated technologies described within this collection of publications are testament to the current trend in healthcare education. SBE through VR-based training (VRT) was the theme of two publications under this research topic (Mohamad et al, Favier et al), whilst another group described the use of augmented reality (AR) (Cofano et al) in a prospective case series that involved telementoring, hologram and 3D reconstruction in spinal surgery. The use of AR and VR was propagated during the pandemic that imposed huge limitations and obstacles to face-to-face physical classroom teaching-learning activities throughout 2020–2022. Adapting educational materials and adopting online or blended approaches to maintain training and certification during these clinically challenging times was imperative to ensure a sustainable workforce across the globe. Mohamad et al highlighted the development of VRT according to an effective ontology design, offering a different and useful perspective and direction for those wishing to incorporate VRT into the healthcare domain. The authors proposed a solid knowledge base be established to enable a comprehensive strategy for the development of VRT specifically the design of future VR applications especially in the healthcare domain.

Haptic technology in VRT improved the way in which surgical training could be delivered and was seen as a game-changer in surgical-based training, now and for decades to come (Favier et al). The introduction of haptics in VRT produced more immersive training opportunities in many surgical-based fields, compared with traditional training methods. Previously, most surgical training was conducted on patients, leaving very little room for error. The introduction of haptic technology in VRT allowed trainees to hone their surgical skills more quickly through repetitive, flexible and individualized training without fear of making mistakes, potentially shortening the learning curve of a surgeon.

During the COVID-19 pandemic, clinical teaching and the usual classroom-based continuing-medical-education (CME) sessions were significantly disrupted. Medical students in most countries could not access clinical areas such as wards, operating theatres, and emergency rooms, leading most clinician-educators to turn to online teaching approaches through telesimulation and video-based learning. Even though students still preferred the face-to-face approach (Saiboon et al), remote online learning became the lifeline to clinical teaching through an online CME approach (Schulte et al). For simple cognitive learning, like teaching disaster response medicine to preclinical undergraduate medical students, an asynchronous approach was successfully implemented through the **e**-**l**earning **i**n **t**eaching **e**mergency **d**isaster **r**esponse (ELITE DR) module (Saiboon et al). This module allowed flexibility for students to access the teaching material at any time from any place. Skills based teaching as in performing **f**ocused **a**ssessment with **s**onography in **t**rauma (FAST) was conducted successfully through the self-instructional-video (SIV) approach (Isa et al). The authors demonstrated that SIV teaching was not inferior to face-to-face classroom teaching. In fact, training of a more complex surgical procedure like sinus surgery was successfully conducted online using a combination of telesimulation, web-conferencing and task trainer (using 3-D printed sinus models) together with remote supervision and feedback from the subject matter expert (Suzuki et al). Apart from simple cognitive and psychomotor skills training, interactive video usage also promoted teaching of higher order thinking and teamwork, and enhanced the perception of authenticity during online training (Musa et al).

Another important point to emphasize in relation to online video-based learning was the benefit of preparatory educational video material to be viewed before the actual teaching session. This exposure promoted students’ curiosity level and correlated positively with subsequent learning and understanding of the topic (Ho et al). In addition, educational videos played a vital role in patient education by reducing preoperative anxiety among parents and their children who were scheduled for surgery (Härter et al).

In summary, it has been a rewarding journey indeed for all four guest editors of this research topic. The project has reaffirmed our belief in simulation as a universally accepted educational modality; the included articles are proof that simulation is adaptable to online platforms and applies across geographical boundaries. Newer cutting-edge technologies offer added and flexible dimensions to the learning experience which, going forward, are likely to be adopted as a matter of routine, judging by the rapidity of development of applications for these modalities in SBE. The beginning has been great; it is now time to carry the journey forward where, based on the level of interest, an entire Frontiers journal on simulation and online learning may emerge, reflected by the level of interest from this current endeavour.

